# Supplementary material for: Advantageous and disadvantageous inequality aversion can be taught through learning of others’ preferences
Source: eLife. 2025 Dec 11;14:RP102800. doi: 10.7554/eLife.102800 (PMC12698084; doi:10.7554/eLife.102800)
Supplement: Supplementary file 1. [file elife-102800-supp1.docx]

**Supplementary Information**

**Table A.** Reinforcement rates governing the Teacher’s feedback in the Learning Phase. For example, a rate 90% for 90:10 offers indicates that on 90% of trials with that offer type, the Teacher indicated they would have preferred rejection of that offer.

| Offer type | Adv-Dis-I | Dis-I |
| --- | --- | --- |
| 90:10 | 90% | 90% |
| 70:30 | 75% | 75% |
| 50:50 | 0% | 0% |
| 30:70 | 75% | 0% |
| 10:90 | 90% | 0% |

**Table B.** Fairness rating of the Teacher in the Learning Phase in Experiment 1. The teacher rated 90:10 offers as Strongly unfair (1) or Unfair (2) randomly in both conditions.

| Offer type | Adv-Dis-I | Dis-I |
| --- | --- | --- |
| 90:10 | 1 or 2 | 1 or 2 |
| 70:30 | 2 or 3 | 2 or 3 |
| 50:50 | 6 or 7 | 6 or 7 |
| 30:70 | 2 or 3 | 6 or 7 |
| 10:90 | 1 or 2 | 6 or 7 |

**Table C.** Baseline Phase Choice Behavior in Experiment 1. Linear mixed model coefficients (fixed effects) indicating effects of condition and Offer type on the baseline Rejection rate.

Model:

$$RejectionRate \sim0+(DisI+AdvDisI):(Offer10+Offer30+Offer50+Offer70+Offer90)+(1|Subject)$$

| Predictor | Estimate | SE | t | p |
| --- | --- | --- | --- | --- |
| Adv-Dis-I×Offer 90:10 | 0.67 | 0.03 | 23.29 | <0.001 |
| Adv-Dis-I×Offer 70:30 | 0.34 | 0.03 | 11.85 | <0.001 |
| Adv-Dis-I×Offer 50:50 | 0.03 | 0.03 | 0.98 | 0.329 |
| Adv-Dis-I×Offer 30:70 | 0.05 | 0.03 | 1.60 | 0.109 |
| Adv-Dis-I×Offer 10:90 | 0.08 | 0.03 | 2.86 | 0.004 |
| Dis-I×Offer 90:10 | 0.54 | 0.03 | 18.69 | <0.001 |
| Dis-I×Offer 70:30 | 0.25 | 0.03 | 8.85 | <0.001 |
| Dis-I×Offer 50:50 | 0.03 | 0.03 | 1.05 | 0.296 |
| Dis-I×Offer 30:70 | 0.06 | 0.03 | 1.95 | 0.051 |
| Dis-I×Offer 10:90 | 0.09 | 0.03 | 3.14 | 0.002 |

**Table D.** Baseline Phase Rating Behavior in Experiment 1. Linear mixed model coefficients (fixed effects) indicating effects of condition and Offer type on the baseline Fairness Ratings (testing if the coefficient is equal to 4).

The models are the same as in Table C, except for that the dependent variable is the fairness rating.

| Predictor | Estimate | SE | t | p |
| --- | --- | --- | --- | --- |
| Adv-Dis-I×Offer 90:10 | 2.06 | 0.14 | -13.92 | <0.001 |
| Adv-Dis-I×Offer 70:30 | 3.16 | 0.14 | -5.99 | <0.001 |
| Adv-Dis-I×Offer 50:50 | 5.22 | 0.14 | 8.71 | <0.001 |
| Adv-Dis-I×Offer 30:70 | 3.92 | 0.14 | -0.55 | 0.583 |
| Adv-Dis-I×Offer 10:90 | 3.19 | 0.14 | -5.78 | <0.001 |
| Dis-I×Offer 90:10 | 2.22 | 0.14 | -12.75 | <0.001 |
| Dis-I×Offer 70:30 | 3.24 | 0.14 | -5.42 | <0.001 |
| Dis-I×Offer 50:50 | 5.04 | 0.14 | 7.42 | <0.001 |
| Dis-I×Offer 30:70 | 4.08 | 0.14 | 0.55 | 0.583 |
| Dis-I×Offer 10:90 | 3.45 | 0.14 | -3.96 | <0.001 |

**Table E.** Contagion effects in rejection rates in Experiment 1, computed as the difference between Transfer and Learning Phase rejection rates. Linear mixed model coefficients (fixed effects) indicating the effects of Condition and Offer Type on the Rejection rate changes from Baseline to Transfer phase.

The model is the same as in Table C, except for that the dependent variable is rejection rates changes from baseline to transfer.

| Predictor | Estimate | SE | t | p |
| --- | --- | --- | --- | --- |
| Adv-Dis-I-Averse×Offer 90:10 | 0.14 | 0.03 | 4.97 | <0.001 |
| Adv-Dis-I-Averse×Offer 70:30 | 0.12 | 0.03 | 4.25 | <0.001 |
| Adv-Dis-I-Averse×Offer 50:50 | 0.03 | 0.03 | 0.94 | 0.349 |
| Adv-Dis-I-Averse×Offer 30:70 | 0.09 | 0.03 | 3.10 | 0.002 |
| Adv-Dis-I-Averse×Offer 10:90 | 0.12 | 0.03 | 4.47 | <0.001 |
| Dis-I-Averse×Offer 90:10 | 0.18 | 0.03 | 6.56 | <0.001 |
| Dis-I-Averse×Offer 70:30 | 0.14 | 0.03 | 5.12 | <0.001 |
| Dis-I-Averse×Offer 50:50 | 0.02 | 0.03 | 0.65 | 0.517 |
| Dis-I-Averse×Offer 30:70 | -0.01 | 0.03 | -0.43 | 0.666 |
| Dis-I-Averse×Offer 10:90 | -0.03 | 0.03 | -1.08 | 0.280 |
| **Contrasts between conditions:** |  |  |  |  |
| Adv-Dis-I×Offer 90:10 – Dis-I×Offer 90:10 | -0.04 | 0.04 | -1.12 | 0.263 |
| Adv-Dis-I×Offer 70:30 – Dis-I×Offer 70:30 | -0.02 | 0.04 | -0.61 | 0.541 |
| Adv-Dis-I×Offer 50:50 – Dis-I×Offer 50:50 | 0.01 | 0.04 | 0.20 | 0.839 |
| Adv-Dis-I×Offer 30:70 – Dis-I×Offer 30:70 | 0.10 | 0.04 | 2.50 | 0.013 |
| Adv-Dis-I×Offer 10:90 – Dis-I×Offer 10:90 | 0.15 | 0.04 | 3.92 | <0.001 |

**Table F.** Contagion effects in Fairness ratings in Experiment 1, computed as the difference between Transfer and Learning Phase fairness ratings. Linear mixed model coefficients (fixed effects) indicating the effects of Condition and Offer Type on the Fairness rating changes.

The model is the same as in Table C, except for that the dependent variable is the fairness rating changes from baseline to transfer.

| Predictor | Estimate | SE | t | p |
| --- | --- | --- | --- | --- |
| Adv-Dis-I×Offer 90:10 | -0.14 | 0.12 | -1.15 | 0.252 |
| Adv-Dis-I×Offer 70:30 | -0.28 | 0.12 | -2.32 | 0.021 |
| Adv-Dis-I×Offer 50:50 | 0.05 | 0.12 | 0.44 | 0.662 |
| Adv-Dis-I×Offer 30:70 | -0.54 | 0.12 | -4.42 | <0.001 |
| Adv-Dis-I×Offer 10:90 | -0.76 | 0.12 | -6.23 | <0.001 |
| Dis-I×Offer 90:10 | -0.06 | 0.12 | -0.49 | 0.623 |
| Dis-I×Offer 70:30 | -0.07 | 0.12 | -0.57 | 0.566 |
| Dis-I×Offer 50:50 | 0.25 | 0.12 | 2.05 | 0.041 |
| Dis-I×Offer 30:70 | 0.32 | 0.12 | 2.59 | 0.010 |
| Dis-I×Offer 10:90 | 0.28 | 0.12 | 2.29 | 0.022 |
| **Contrasts between conditions:** |  |  |  |  |
| Adv-Dis-I×Offer 90:10 – Dis-I×Offer 90:10 | -0.08 | 0.17 | -0.46 | 0.643 |
| Adv-Dis-I×Offer 70:30 – Dis-I×Offer 70:30 | -0.21 | 0.17 | -1.24 | 0.217 |
| Adv-Dis-I×Offer 50:50 – Dis-I×Offer 50:50 | -0.20 | 0.17 | -1.14 | 0.255 |
| Adv-Dis-I×Offer 30:70 – Dis-I×Offer 30:70 | -0.86 | 0.17 | -4.96 | <0.001 |
| Adv-Dis-I×Offer 10:90 – Dis-I×Offer 10:90 | -1.04 | 0.17 | -6.02 | <0.001 |

**Table G.** Mixed-effects logistic regression examining Rejection choices (Reject vs. Accept) during the Learning Phase in Experiment 1, as a function of the interactions between Condition, Offer Type, and Trial Number.

Model:

$$Rejection Choice \sim(AdvDisI+DisI):(Offer10+Offer30+Offer50+Offer70+Offer90)*ZTrial - ZTrial - 1 + ((Offer10+Offer30+Offer50+Offer70+Offer90)*ZTrial-1-ZTrial||Subject)$$

| Predictor | Estimate | SE | p |
| --- | --- | --- | --- |
| Adv-Dis-I×Offer 90:10 | 5.22 | 0.55 | <0.001 |
| Adv-Dis-I×Offer 70:30 | 0.11 | 0.29 | 0.703 |
| Adv-Dis-I×Offer 50:50 | -5.55 | 0.41 | <0.001 |
| Adv-Dis-I×Offer 30:70 | -2.72 | 0.35 | <0.001 |
| Adv-Dis-I×Offer 10:90 | -1.38 | 0.41 | <0.001 |
| Dis-I×Offer 90:10 | 4.57 | 0.53 | <0.001 |
| Dis-I×Offer 70:30 | -0.23 | 0.29 | 0.428 |
| Dis-I×Offer 50:50 | -5.50 | 0.42 | <0.001 |
| Dis-I×Offer 30:70 | -5.86 | 0.46 | <0.001 |
| Dis-I×Offer 10:90 | -6.34 | 0.56 | <0.001 |
| Adv-Dis-I×Offer 90:10×Trial | 0.10 | 0.21 | 0.627 |
| Adv-Dis-I×Offer 70:30×Trial | 0.30 | 0.09 | 0.001 |
| Adv-Dis-I×Offer 50:50×Trial | 0.15 | 0.16 | 0.327 |
| Adv-Dis-I×Offer 30:70×Trial | 0.47 | 0.11 | <0.001 |
| Adv-Dis-I×Offer 10:90×Trial | 0.71 | 0.17 | <0.001 |
| Dis-I×Offer 90:10×Trial | 0.64 | 0.21 | 0.002 |
| Dis-I×Offer 70:30×Trial | 0.30 | 0.09 | 0.001 |
| Dis-I×Offer 50:50×Trial | 0.06 | 0.16 | 0.719 |
| Dis-I×Offer 30:70×Trial | -0.31 | 0.18 | 0.085 |
| Dis-I×Offer 10:90×Trial | -0.32 | 0.27 | 0.237 |
| **Contrasts of Interested:** |  |  |  |
| Adv-Dis-I×Offer 90:10×Trial – Dis-I×Offer 90:10×Trial | -0.48 | 0.21 | 0.593 |
| Adv-Dis-I×Offer 70:30×Trial – Dis-I×Offer 70:30×Trial | -0.01 | 0.14 | 0.150 |
| Adv-Dis-I×Offer 50:50×Trial– Dis-I×Offer 50:50×Trial | 0.00 | 0.21 | 0.086 |
| Adv-Dis-I×Offer 30:70×Trial – Dis-I×Offer 30:70×Trial | 0.77 | 0.24 | <0.001 |
| Adv-Dis-I×Offer 10:90×Trial– Dis-I×Offer 10:90×Trial | 1.10 | 0.33 | <0.001 |

**Table H.** Summary of Model Comparison in Experiment 1.

| **Model** | **nLL** | **AIC** | **Mean Parameter Estimates** | | | | | | |  |
| --- | --- | --- | --- | --- | --- | --- | --- | --- | --- | --- |
| Preference Inference | 3765.02 | 9130.04 | $\alpha_{0}$ | $\beta_{0}$ | $\eta$ | $\tau$ |  |  |  | |
|  |  |  | 2.01 | 1.38 | 0.14 | 0.83 |  |  |  | |
| Static preference | 4146.96 | 9493.93 | $\alpha$ | $\beta$ | $\tau$ |  |  |  |  | |
|  |  |  | 2.01 | 1.58 | 0.67 |  |  |  |  | |
| RL Similarity | 3748.69 | 9497.38 | $\alpha_{0}$ | $\beta_{0}$ | $\eta$ | $\tau$ | $\sigma$ |  |  | |
|  |  |  | 1.29 | 0.50 | 0.10 | 19.85 | 41.30 |  |  | |
| RL Basic | 6519.68 | 13839.36 | $\eta$ | $\tau$ |  |  |  |  |  | |
|  |  |  | 0.28 | 14.27 |  |  |  |  |  | |
| RL Separate Initial | 4097.73 | 10995.45 | $\eta$ | $\tau$ | $V_{1}$ | $V_{2}$ | $V_{3}$ | $V_{4}$ | $V_{5}$ | |
|  |  |  | 0.06 | 35.14 | 0.72 | 0.44 | 0.11 | 0.20 | 0.26 | |
| RL Offer Sensitive | 5393.92 | 13187.84 | $\eta_{1}$ | $\eta_{2}$ | $\eta_{3}$ | $\eta_{4}$ | $\eta_{5}$ | $\tau$ |  | |
|  |  |  | 0.35 | 0.16 | 0.74 | 0.44 | 0.48 | 33.68 |  | |
| Random Choosing | 4393.93 | 10787.85 | $p_{1}$ | $p_{2}$ | $p_{3}$ | $p_{4}$ | $p_{5}$ |  |  | |
|  |  |  | 0.80 | 0.50 | 0.05 | 0.16 | 0.24 |  |  | |

Parameters:

$\alpha, \alpha_{0}$: Envy parameters

$\beta, \beta_{0}$: Guilt parameters

$\eta, \eta_{i}(i=1,2,3,4,5)$: Learning rates

$\tau$: Inverse temperature

$\sigma$: Standard deviation of the normal distribution

$V_{i}(i=1,2,3,4,5)$: Initial values

$p_{i}(i=1,2,3,4,5)$: Random choosing probabilities

**Table I.** Contagion effects in Experiment 2. Linear mixed model coefficients (fixed effects) indicating the effects of Condition and Offer Type on the Rejection rate changes (from Baseline phase to Transfer phase). This model specification is identical to that used in Experiment 1 (reported in Table C).

| Predictor | Estimate | SE | t | p |
| --- | --- | --- | --- | --- |
| Adv-Dis-I×Offer 90:10 | 0.13 | 0.03 | 4.40 | <0.001 |
| Adv-Dis-I×Offer 70:30 | 0.11 | 0.03 | 3.78 | <0.001 |
| Adv-Dis-I×Offer 50:50 | 0.05 | 0.03 | 1.64 | 0.102 |
| Adv-Dis-I×Offer 30:70 | 0.03 | 0.03 | 1.02 | 0.307 |
| Adv-Dis-I×Offer 10:90 | 0.06 | 0.03 | 1.96 | 0.050 |
| Dis-I×Offer 90:10 | 0.18 | 0.03 | 6.00 | <0.001 |
| Dis-I×Offer 70:30 | 0.09 | 0.03 | 3.02 | 0.003 |
| Dis-I×Offer 50:50 | -0.02 | 0.03 | -0.68 | 0.495 |
| Dis-I×Offer 30:70 | -0.03 | 0.03 | -1.07 | 0.283 |
| Dis-I×Offer 10:90 | -0.06 | 0.03 | -2.10 | 0.036 |
| **Contrasts between conditions** |  |  |  |  |
| Adv-Dis-I×Offer 90:10 – Dis-I×Offer 90:10 | -0.05 | 0.04 | -1.13 | 0.259 |
| Adv-Dis-I×Offer 70:30 – Dis-I×Offer 70:30 | 0.02 | 0.04 | 0.54 | 0.590 |
| Adv-Dis-I×Offer 50:50 – Dis-I×Offer 50:50 | 0.07 | 0.04 | 1.64 | 0.101 |
| Adv-Dis-I×Offer 30:70 – Dis-I×Offer 30:70 | 0.06 | 0.04 | 1.48 | 0.138 |
| Adv-Dis-I×Offer 10:90 – Dis-I×Offer 10:90 | 0.12 | 0.04 | 2.87 | 0.004 |

**Table J.** Contagion effect of Fairness rating in Experiment 2. Linear mixed model coefficients (fixed effects) indicating the effects of Condition and Offer Type on the Fairness rating changes from Baseline to Transfer phase.

The model is the same as in Table D

| Predictor | Estimate | SE | t | p |
| --- | --- | --- | --- | --- |
| Adv-Dis-I×Offer 90:10 | -0.23 | 0.13 | -1.78 | 0.076 |
| Adv-Dis-I×Offer 70:30 | -0.41 | 0.13 | -3.20 | 0.001 |
| Adv-Dis-I×Offer 50:50 | -0.24 | 0.13 | -1.91 | 0.056 |
| Adv-Dis-I×Offer 30:70 | -0.53 | 0.13 | -4.22 | <0.001 |
| Adv-Dis-I×Offer 10:90 | -0.84 | 0.13 | -6.59 | <0.001 |
| Dis-I×Offer 90:10 | -0.29 | 0.13 | -2.28 | 0.023 |
| Dis-I×Offer 70:30 | -0.15 | 0.13 | -1.21 | 0.228 |
| Dis-I×Offer 50:50 | 0.24 | 0.13 | 1.88 | 0.060 |
| Dis-I×Offer 30:70 | 0.09 | 0.13 | 0.68 | 0.498 |
| Dis-I×Offer 10:90 | 0.14 | 0.13 | 1.10 | 0.272 |
| **Contrasts between conditions** |  |  |  |  |
| Adv-Dis-I×Offer 90:10 – Dis-I×Offer 90:10 | 0.06 | 0.18 | 0.35 | 0.723 |
| Adv-Dis-I×Offer 70:30 – Dis-I×Offer 70:30 | -0.25 | 0.18 | -1.41 | 0.159 |
| Adv-Dis-I×Offer 50:50 – Dis-I×Offer 50:50 | -0.48 | 0.18 | -2.68 | 0.007 |
| Adv-Dis-I×Offer 30:70 – Dis-I×Offer 30:70 | -0.62 | 0.18 | -3.46 | <0.001 |
| Adv-Dis-I×Offer 10:90 – Dis-I×Offer 10:90 | -0.97 | 0.18 | -5.44 | <0.001 |

**Table K.** Mixed-effects logistic regression examining Rejection choices (Reject vs. Accept) during the Learning Phase in Experiment 2, as a function of the interactions between Condition, Offer Type, and Trial Number.

The model is the same as in Table G.

| Predictor | Estimate | SE | p |
| --- | --- | --- | --- |
| Adv-Dis-I×Offer 90:10 | 2.20 | 0.48 | <0.001 |
| Adv-Dis-I×Offer 70:30 | -0.79 | 0.35 | 0.024 |
| Adv-Dis-I×Offer 50:50 | -3.94 | 0.27 | <0.001 |
| Adv-Dis-I×Offer 30:70 | -3.36 | 0.36 | <0.001 |
| Adv-Dis-I×Offer 10:90 | -2.54 | 0.44 | <0.001 |
| Dis-I×Offer 90:10 | 1.07 | 0.48 | 0.026 |
| Dis-I×Offer 70:30 | -1.32 | 0.35 | <0.001 |
| Dis-I×Offer 50:50 | -4.41 | 0.30 | <0.001 |
| Dis-I×Offer 30:70 | -4.97 | 0.39 | <0.001 |
| Dis-I×Offer 10:90 | -5.24 | 0.50 | <0.001 |
| Adv-Dis-I×Offer 90:10×Trial | 0.26 | 0.13 | 0.049 |
| Adv-Dis-I×Offer 70:30×Trial | 0.16 | 0.08 | 0.053 |
| Adv-Dis-I×Offer 50:50×Trial | 0.21 | 0.12 | 0.080 |
| Adv-Dis-I×Offer 30:70×Trial | 0.17 | 0.10 | 0.102 |
| Adv-Dis-I×Offer 10:90×Trial | 0.39 | 0.17 | 0.019 |
| Dis-I×Offer 90:10×Trial | 0.47 | 0.13 | <0.001 |
| Dis-I×Offer 70:30×Trial | 0.29 | 0.09 | <0.001 |
| Dis-I×Offer 50:50×Trial | -0.12 | 0.14 | 0.378 |
| Dis-I×Offer 30:70×Trial | -0.36 | 0.13 | 0.006 |
| Dis-I×Offer 10:90×Trial | -0.49 | 0.21 | 0.020 |
| **Contrasts of Interest:** |  |  |  |
| Adv-Dis-I×Offer 90:10×Trial – Dis-I×Offer 90:10×Trial | -0.25 | 0.19 | 0.707 |
| Adv-Dis-I×Offer 70:30×Trial – Dis-I×Offer 70:30×Trial | -0.14 | 0.12 | 0.359 |
| Adv-Dis-I×Offer 50:50×Trial– Dis-I×Offer 50:50×Trial | 0.33 | 0.19 | 0.054 |
| Adv-Dis-I×Offer 30:70×Trial – Dis-I×Offer 30:70×Trial | 0.50 | 0.17 | 0.004 |
| Adv-Dis-I×Offer 10:90×Trial– Dis-I×Offer 10:90×Trial | 0.81 | 0.26 | 0.002 |

**Table L.** Summary of Model Comparison in Experiment 2.

| **Model** | **nLL** | **AIC** | **Mean Parameter Estimates** | | | | | | |  |
| --- | --- | --- | --- | --- | --- | --- | --- | --- | --- | --- |
| Preference Inference | 6250.45 | 14100.90 | $\alpha_{0}$ | $\beta_{0}$ | $\eta$ | $\tau$ |  |  |  | |
|  |  |  | 1.75 | 1.48 | 0.07 | 0.82 |  |  |  | |
| Static preference | 6522.31 | 14244.63 | $\alpha$ | $\beta$ | $\tau$ |  |  |  |  | |
|  |  |  | 1.74 | 1.53 | 0.78 |  |  |  |  | |
| RL Sim | 6130.80 | 14261.60 | $\alpha_{0}$ | $\beta_{0}$ | $\eta$ | $\tau$ | $\sigma$ |  |  | |
|  |  |  | 1.10 | 0.51 | 0.08 | 18.21 | 44.58 |  |  | |
| RL Separate Initial | 6720.48 | 16240.97 | $\eta$ | $\tau$ | $V_{1}$ | $V_{2}$ | $V_{3}$ | $V_{4}$ | $V_{5}$ | |
|  |  |  | 0.01 | 38.16 | 0.58 | 0.39 | 0.15 | 0.23 | 0.26 | |
| Random Choosing | 6894.55 | 15789.11 | $p_{1}$ | $p_{2}$ | $p_{3}$ | $p_{4}$ | $p_{5}$ |  |  | |
|  |  |  | 0.62 | 0.43 | 0.08 | 0.15 | 0.22 |  |  | |

Parameters:

$\alpha, \alpha_{0}$: Envy parameters

$\beta, \beta_{0}$: Guilt parameters

$\eta$: Learning rates

$\tau$: Inverse temperature.

$V_{i}(i=1,2,3,4,5)$: Initial values

$p_{i}(i=1,2,3,4,5)$: Random choosing probabilities
